# Observations upon the Natives of the Hawaiian Isles

**Published:** 1876-09

**Authors:** J. M. Whitney


### DENTAL REGISTER.

VOL. XXX.] SEPTEMBER, 1876. [NO. 9.

### OBSERVATIONS UPON THE NATIVES OF THE HAWAIIAN ISLES.

BY J. M. WHITNEY, D. D. S.

If my apprehensions of the science of dentistry are correct, it has to do with a wider range of knowledge than the elimination of the few propositions that first present themselves to the dental student.

The corollaries from these few great propositions reach out to interlace with all truth; therefore I shall need to make no apology for taking a few moments in giving you some observations made in a foreign land, which I trust may not be without some interest or value.

When the Hawaiian Islands were first discovered by Captain Cook, less than a century ago, he estimated their population to be between 400,000 and 600,000 persons.

At present there are but 48,000. This fearful decrease in number is largely due to the venereal poison introduced by the licentious crew of Captain Cook, and extended by many other similar crews that have touched those shores since that time.

From what our journals have frequently told us, we should expect to see very marked signs of this poisoned life as inheritances in the teeth of this people; but though my observations were careful and extended I saw very little of that saw-teeth condition so frequently mentioned.

I looked long, but in vain, through those extensive fields of bleached bones to find traces upon teeth or palatine arch of that disease that had surely gone down with each of them.

You can get from the varied specimens I have brought before you to-day, a good idea of what careful research could do for the theory of the effects of syphilis upon the teeth.

Yet its influence upon the system in general was marked, and doubtless was one of the main factors in the more rapid decay of the teeth of the later generations.

The teeth of the average native are good, though by no means as perfect as our text books and instructors would have us suppose. I never had the opportunity of examining the teeth of a native without finding more or less decay.

When I first saw them I was surprised to see so many of the older ones without front lower teeth, and very naturally concluded that they had been lost through the effects of salivary calculi. Upon examination, I found little or none of this deposit, but the teeth were broken off just at the gums. Upon enquiry, I learned that it had been their custom, on the death of their chief or king, or one to whom they were greatly attached, to manifest their grief by knocking out their teeth. Certainly a very unique and permanent mode of wearing mourning.

While incidentally touching the subject of salivary calculi, I would like to show you this specimen which, for amount of accretion and serious ravages done, surpasses anything I ever saw before or have seen since.

I believe that had one of our eastern brethren seen this specimen, before he wrote his article upon the benign effects of salivary calculi, it might have modified his views.

That you may appreciate the rare advantages that one has for gathering osteological specimens in that distant country, allow me to ramble a little from my subject and give you their mode of disposing of their dead in the different ages of their history.

On some of the islands there are mountains reaching such an elevation that the air is so rare and pure that bodies do not decompose, but become mummies.

In the olden times the caves upon these mountains were a favorite place of burial, and here to-day may be found as perfect a mummy wrapped in only two or three folds of the native cloth, called tapa, as can be dug from the catacombs of Egypt.

I noticed with great interest that in those early days, long before Cook brought his devastating plague, or markedly acid fruits were introduced, the teeth were nearly perfect. Irregularities and wearing away of the teeth by their coarse food, seemed to be the principal causes for their dental troubles.

In later times, as the people became more degenerate, they preferred to bury their dead in the easily excavated sand by the sea shore rather than carry them to the top of high mountains or scale the perpendicular sides of rocks to deposit them in the almost inaccessible caves that fill them.

Though the latter mode may have shown their degeneracy it was a great convenience to the dental student who, by the key modern dental science has placed in his hands, would unlock and read for the interest, and it is hoped for the good of the present generation, those indellible hieroglyphics written by suffering and unaided humanity centuries before he was born.

Into a hole three or four feet deep, dug in the sand, was placed the body in a sitting posture, with the knees drawn up to the chest and the arms locking the knees.

The shifting winds of time have exposed for miles these whitened skeletons, and you have but to walk, "observe, compare and reflect," until weariness and not flagging interest compels you to turn aside.

In comparing the teeth of these of later generations, with those first mentioned, I found caries much more frequent, in fact, very few were found when the individual had reached middle life in which it did not manifest itself in one or more teeth. I noticed that in the large majority of persons who had reached old age, caries had attacked the labial surfaces of both upper and lower teeth, cutting them off just above the gum. Many were edentulous from this cause long before their death.

That they knew the pangs of dental abscess, these specimens clearly reveal, as also the long drawn out agony of a maldirected dens sapientia.

In this specimen we see the bad effects of retaining the temporary tooth beyond its appointed time.

I have brought to your attention a collection of facts many of which being seen are indisputable. Our time will be lost unless we can draw from them certain correct conclusions that shall aid us in the great work we have undertaken to perform, and through us help our fellow men, whose interest we to-day meet to advance.

Each. for himself, will draw his own inferences, and reach his own conclusions, and while these may differ widely from one another, we have the satisfaction of knowing that so much of truth as we hold will live while the error will be as the dust that clothed these dry bones, blown into the sea of forgetfulness by the sharp winds of time.

The peculiar circumstances that have surrounded these simple people, from the time they first drifted by southern winds upon these islands to the present time, place them in a more favorable situation for us to read the effects of certain diseases and courses of diet than any other people of which we know.

In the oldest races, those buried in the caves, venereal diseases were wholly unknown.

Their diet consisted of a sour paste, made by baking, mashing and allowing to ferment a root called tard, in appearance somewhat resembling our sugar beet, and using the same vegetable, without fermentation, together with pork, fish and fruits. In later times they made use of the same diet with the addition of beef, mutton, lemons, oranges, tamarinds, straw berries and several other markedly acid fruits introduced by foreigners.

As their mode of living and articles of diet are the same, with the exception of beef, mutton and the acid fruits, now as in the times first recorded, it is most natural to conclude that the great change noticeable in their teeth must be due to the introduction of a disease among them that impaired their vital forces, and the chemical effects of the fruit acids. I can see no other causes that were in operation.

It could not be otherwise than that the poison introduced into their systems, together with that handed down to them from the generations passed, had greatly impaired their power to assimilate the proper amount and quality of aliment to sustain the system at large, and the bones and teeth in particular. Therefore the teeth the more readily yielded to the strong fruit acids that had been introduced by the foreigners, and we find as we had reason to expect that the teeth of those buried within the last one hundred years showed a great degeneracy over those of earlier generation.

As an illustration of the effects of those acids, it was a thing of common occurrence to carefully examine and fill all cavities of a child, and in three or four months after the season for tamarinds had passed to find that caries had made attacks at several points.

The effect of eating tamarinds and limes was so marked that unprofessional eyes could note the change, and parents who could control their children were careful to forbid their use.

These observations made upon the living, and upon the white fields of the dead, have lead me to place little confidence in any other theory for dental caries than that of chemical action.

If disease has so impaired the vital force that a sufficient amount and quality of aliment have not been furnished to the teeth, they will the more readily succumb to the chemical forces brought against them, for the same reason that a piece of porous sugar will more readily dissolve than a lump of similar size after it has been crystallized.

I examined with much interest the teeth of those from the Coral Islands of the South Seas, two or three thousand miles south west of the S. Islands where their diet is very limited and miserable, consisting of little else than bread fruit and fish.

Without an exception they were found very defective in form and durability, having that white, opaque look that we not unfrequently meet here.

The natives, as also the missionaries from those islands, say tooth ache is very prevalent among the people.

We certainly can not charge hot bread, mince pies, sugar and condiments with spoiling those teeth.

Do we not see in this people a striking illustration of the effects of a low unnutritious diet upon the teeth?

All agree they are from the same stock as the Hawaiians, living in the same primitive manner, and in the same climate, yet with teeth many degrees below them in point of form and power of resisting unfavorable influences.

Had we no other argument to furnish than this we could with safety press home upon our patients and patrons the necessity of a plain nutritious diet if they would secure well formed and enduring teeth.

One thing is very noticeable in all that race of people, exposure of a nerve of a tooth never seems to give them much trouble. Whenever I was sought to give relief it was invariably for periostitis or dental abscess. I know no reason, unless it is the absence of excessive nervous sensibility.

A practice of several years in that equitable climate convinced me that the dentist in this climate, especially that near those lakes, is often blamed if not cursed for what the weather does rather than what he does, or failed to do. I remember but one case where I had periostitis after filling the root.

Dental abscess yielded to treatment much more readily than here. Fillings near the nerve were not as liable to cause periostitis or death of the pulp. It may be a crumb of comfort to us to fall back upon our dignity, and tell our patients to charge their troubles to their proper sources.

